# Characterization and expression of domains of Alphaherpesvirus bovine 1/5 envelope glycoproteins B in *Komagataella phaffi*

**DOI:** 10.1186/s12917-023-03590-8

**Published:** 2023-01-31

**Authors:** Juan Sebastián Quintero Barbosa, Heidy Yohana Triana Rojas, Janneth Gonzalez, Angela Johana Espejo-Mojica, Carlos Javier Alméciga Díaz, María Fernanda Gutierrez

**Affiliations:** 1 Virology Laboratory, Infectious Diseases Group, Microbiology Department, Faculty of Science Pontificia, Universidad Javeriana, Bogotá, D.C Colombia; 2 Institute for the Study of Inborn Errors of Metabolism, Faculty of Science, Pontificia Universidad Javeriana, Bogotá, D.C Colombia; 3 Nutrition and Biochemistry Department, Faculty of Science, Pontificia Universidad Javeriana, Bogotá, D.C Colombia

**Keywords:** Alphaherpesvirus bovine, Vaccine, Recombinant protein, IBR

## Abstract

**Background:**

*Bovine herpes virus* (BoHV 1 and BoHV-5) are the causative agents of infectious bovine rhinotracheitis (IBR). IBR is responsible for important economic losses in the cattle industry. The envelope glycoprotein B (gB) is essential for BoHV infection of cattle's upper respiratory and genital tract. gB is one of the main candidate antigens for a potential recombinant vaccine since it induces a strong and persistent immune response.

**Results:**

In this study, gB of BoHV-1 and BoHV-5 was characterized in terms of function, structure, and antigenicity through bioinformatics tools. gB showed conserved sequence and structure, so, both domains named PH Like 1 and 2 domains of each virus were selected for the design of a bivalent vaccine candidate. The immunoinformatic study showed that these two domains have epitopes recognizable by B and T lymphocytes, followed by this, the cDNA domains from BoHV-1/5 gB (Domains-gB) were transformed into the yeast *Komagataella phaffii* GS115 (previously known as *Pichia pastoris)*. A recombinant protein with molecular weight of about 110 kDa was obtained from the culture media. The vaccine candidate protein (Domains-gB) was recognized by a monoclonal antibody from a commercial ELISA kit used for IBR diagnostic, which may suggest that the epitopes are conserved of the entire infectious virus.

**Conclusion:**

Overall, it was shown that the recombinant domains of BoHV-1/5 gB have antigenic and immunogenic properties similar to the native gB. This vaccine candidate is promising to be used in future studies to assess its immunogenicity in an animal model.

**Supplementary Information:**

The online version contains supplementary material available at 10.1186/s12917-023-03590-8.

## Background

*Bovine herpes virus* (BoHV) 1 and 5 are viruses belonging to the family *Herpesviridae*, subfamily *alphaherpesvirinae* and genus *varicellovirus* [1]. BoHV1/5 are the causative agents of the infectious bovine rhinotracheitis (IBR) and have tropism for the upper respiratory tract and genital tract cells [2]. IBR is characterized by purulent rhinorrhea, conjunctivitis, fever, prostration and lack of pustular vulvovaginitis, balanoposthitis, abortions, and in some cases, neurological disease. Particularly for these viruses, after the primary infection is solved and non-clinical signs are seen in the animals, the viral infection persists throughout the individual's life in a state of latency in trigeminal or sacral nervous ganglia, causing permanent infections in cattle herds [3].

The structural components of BoHV-1/5 include a double-stranded DNA genome into an icosahedral nucleocapsid surrounded by the tegument, an amorphous layer with some structured regions. Finally, it is covered with a lipoprotein envelope that contains ten important glycoproteins for the viral cycle [1].

Currently, there are several vaccine alternatives, including traditional (inactivated and attenuated vaccines) and new generation vaccines (recombinant, vectors, ADN) [4]. Although World Organization for Animal Health (OIE) reports that vaccination against BoHV-1/5 can effectively reduce clinical manifestations and economic losses, the current vaccines do not completely protect against infection, latency, and virus reactivation [4]. Therefore, new vaccine technologies against these herpesviruses are required.

Recent reports on the development of vaccine candidates have been focused on the glycoproteins due to their location (i.e., envelope) and importance in the viral cycle [5–7]. Particularly, BoHV-1 and BoHV-5 have the same amount of glycoproteins and share a high degree of structural and functional homology [8, 9]. Herpesviruses glycoproteins are responsible for the initial adhesion of the virus to cellular receptors and the subsequent penetration into the host cells, influencing cell tropism [10–12]. In addition, glycoproteins participate in several processes such as capsid wrapping, viral release, and transmission of infection through a cell-to-cell dissemination process [10, 11, 13].

The 10 glycoproteins (gB, gC, gD, gE, gG, gH, gI, gK, gL and gM) encoded by the BoHVs genomes, are in the outer layer of the viral particle [11, 14–18]. Due to their location, most of them are recognized by the host´s immune system producing antibodies against these proteins. However, antibodies directed to gB, gC and gD have been found at a higher titer compared to the other glycoproteins, probably because these proteins can be found both on the viral envelope and on the surface of infected cells [8, 9, 19]. These characteristics make these glycoproteins potential candidates for vaccination against BoHV-1/5 [6]. Some researchers have proposed different vaccine candidates against BoHV-1 or BoHV-5 using glycoprotein D, showing better results than the commercial inactivated virus vaccine but have not been able to prevent neuroinvasion and viral reactivation [6, 7].

BoHV-1/5 glycoprotein B (gB) plays an essential role in the first steps of the viral cycle, such as adhesion [20–22], facilitating the penetration and subsequent stages of the cycle. Previous studies have shown immune responses directed to this specific glycoprotein [21]. In this sense, stimulating the production of neutralizing antibodies against gB may help in further prevention for subsequent viral stages, such as latency and reactivation [7]. To the best of our knowledge, this is the first time that gB domains for both viruses are characterized, studied through inmuno-informatics, and tested as within a bivalent vaccine candidate against BoHV-1/5. Thus, the aim of this study was to characterize in silico the function, structure, and immunology profile the gB of BoHV-1 and BoHV-5, as well as to express it in the yeast *Komagataella phaffii* to evaluate immunogenic potential.

## Results

### Characterization of glycoprotein B

Table 1 shows the predicted physicochemical properties of gB from BoHV-1 and BoHV-5. Grand average of hydropathy (GRAVY) was also predicted, showing that the glycoproteins can be hydrophilic since the predicted value was negative, which favoring glycoproteins solubility in water.

**Table 1:** Predicted physicochemical properties of B Glycoproteins of *Bovine Herpes virus* 1 and 5

Physicochemical characteristics																
Glycoprotein	gB		gC		gD		gN		gM		gE		gH		gI	
Species BoHV	1	5	1	5	1	5	1	5	1	5	1	5	1	5	1	5
AA quantity	932	947	521	486	417	417	96	95	438	419	575	599	842	848	380	387
Molecular weight (Kda)	101.19	102.45	55.38	51.44	44.92	44.62	10.26	10.31	45.51	43.17	61.16	63.54	88.37	88.99	39.91	39.67
Theoretical pI	8.46	8.73	8.61	8.97	5.18	5.10	9.77	9.77	10.47	9.81	4.71	4.81	8.66	8.88	10.97	8.60
Total number of negatively charged residues	104	103	51	45	49	47	6	6	18	24	77	82	71	77	24	33
Total number of positively charged residues	109	111	56	53	38	36	11	11	36	35	47	58	77	85	49	38
Instability index	45.86	47.18	57.63	59.80	49.91	50.27	44.12	53.15	38.98	30.77	53.84	53.22	38.49	37.97	65.92	47.79
Aliphatic index	78.56	77.84	68.43	70.99	63.12	68.47	98.65	104.84	114.09	116.06	79.74	72.62	93.61	92.29	81.42	80.75
Average hydropathicity (GRAVY)	(-0.273)	(-0.272)	(-0.352)	(-0.261)	(-0.377)	(-0.286)	0.477	0.558	0.543	0.604	(-0.161)	(-0.311)	0.239	0.212	(-0.029)	0.052
Identity Alignment (%)	91,878		75,287		79,905		78,125		78,475		69,772		85,984		70,951	
Function	Accession		Accession		Penetration		Morphogenesis		Morphogenesis		Cell–cell infection		Cell–cell infection and cell entry		Cell–cell infection	
emPAI abundance	0.778		3,786		0.551				1,683		0.413		0.688		0.389	

**Fig. 1:**
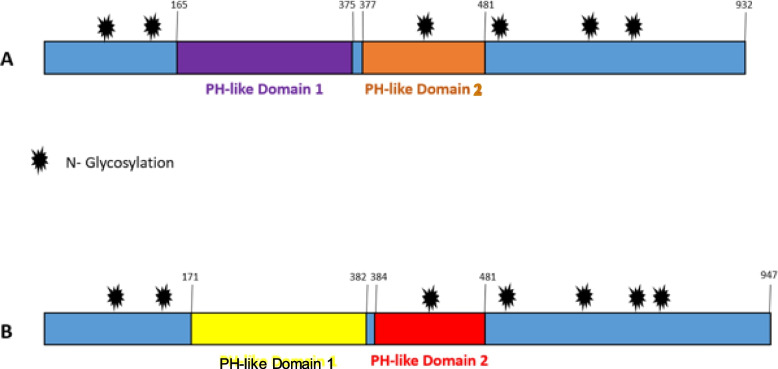
Schematic representation of gB from BoHV-1 and BoHV-5. **A**. gB from of BoHV-1. PH-like domain 1 located between 165 to 375 and PH-like domain 2 located between 377 to 481. Six potential N-glycosylation sites were identified (105, 153, 441, 483, 640 and 706). **B**. gB from BoHV-5. PH-like domain 1 located between 171 to 382 and PH-like domain 2 located between 384 to 481. Seven potential N-glycosylation sites were identified (111, 159, 448, 490, 594, 654 and 720)

In terms of functional annotation, gB from BoHV-1 and BoHV-5 are 932 and 947 amino acids long, respectively. Both gB have two domains located on the surface of the viral particle (PH-like domain 1 and 2). For BoHV-1, the PH-like domain 1 was located between amino acids 165–375; while for BoHV-5 this domain was located between residues 171–382. The second domain was also considered a surface region and was located between residues 377–481 for BoHV1 and 384–481 amino acids for BoHV-5 (Fig. 1). These domains are conserved in all *Alphaherpesvirinae* viruses and play an important role in the viral cycle because they interact with the host cell receptor in the process of viral adhesion. Figure 1A shows the six potential glycosylation sites (residues 105, 153, 441, 483, 640 and 706) of BoHV-1 B glycoprotein and Fig. 1B shows the seven potential glycosylation sites (residues 111, 159, 448, 490, 594, 654 and 720) of BoHV-5 B glycoprotein.

The BoHV-1 gB shares a 91.9% identity in amino acids sequence with the homologous glycoproteins of BoHV-5. Alignment of gB sequences revealed five differences (change amino acids) in the PH-like domain 1; while eight differences between gB were found in the PH-like domain 2.

**Fig. 2:**
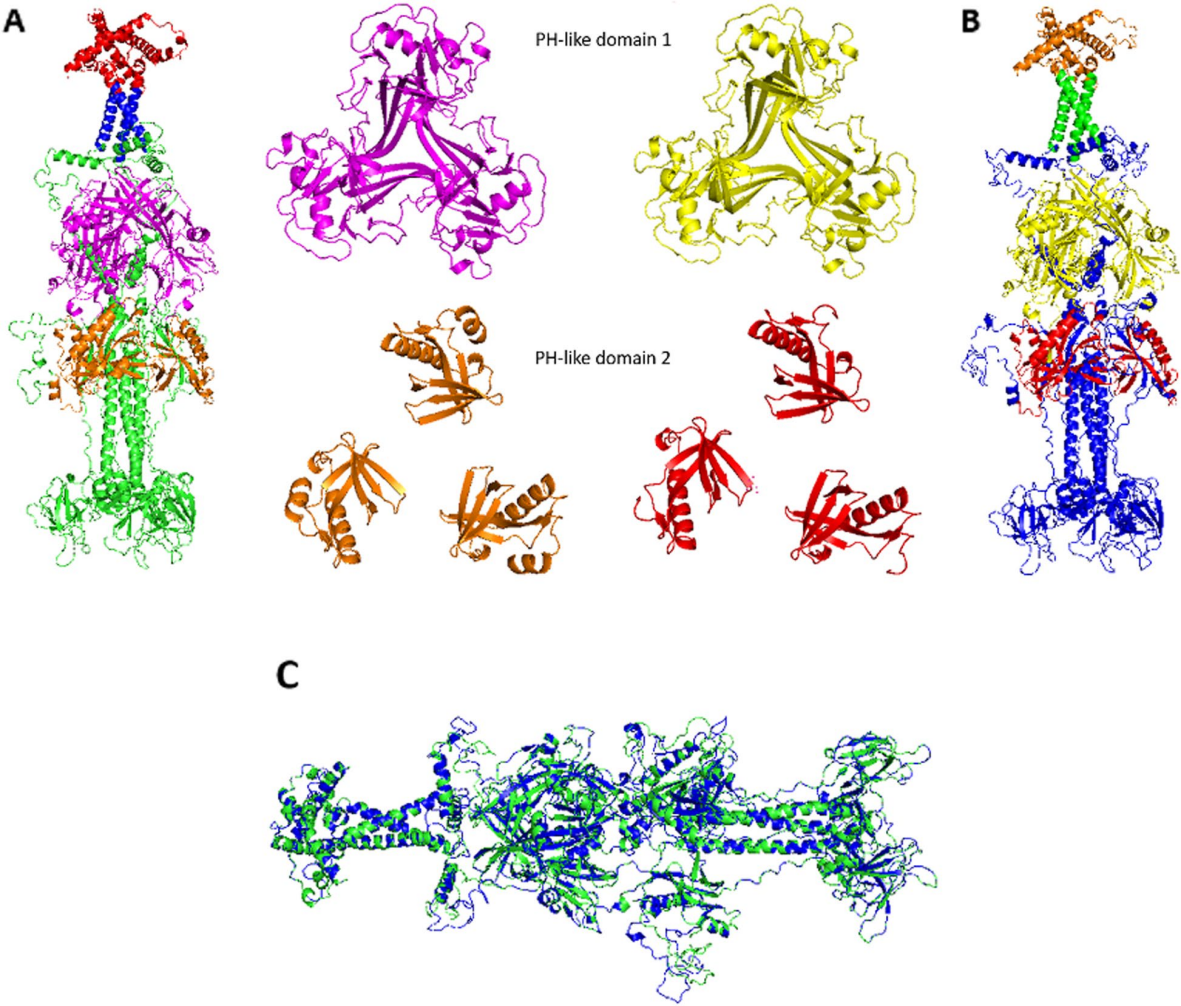
Modelling of gB rom BoHV-1/5. **A**. BoHV-1 gB. Green – overall structure. Magenta—PH-like domain 1. Orange—PH-like domain 2. **B**. BoHV-5 B glycoprotein. Blue – overall structure. Yellow—PH-like domain 1. Red—PH-like domain 2. **C**. Overlapped models of The B glycoprotein model of BoHV-1 (green) overlapping with glycoprotein B model of BoHV-5 (blue)

The predicted secondary structure of BoHV-1 B glycoprotein consists of 21 α-helixes and 41 β-sheets; while for BoHV-5 glycoprotein, 24 α-helixes and 43 β-sheets were predicted. The tertiary model shows a homotrimer conformation in both gB proteins (Fig. 2A). For BoHV-5 the PH-like domain 1 in shown and PH-like domain 2 in red, transmembrane region and intravirion region are shown in green and orange respectively (Fig. 2B). These domains are conserved in the entire Alphaherpesvirinae virus. Figure 2C shows B glycoprotein model of BoHV-1 (green) overlapped with B glycoprotein model of BoHV-5 (blue). These proteins have a high homology structure given by a RMSD value of 0.081 Å. The predicted model for the gB from BoHV-1 by Ramachandran plot showed that 94.27% of the residues were located in the most favored regions. For BoHV-5, the predicted model by Ramachandran plot showed that 94.07% of the residues were located in the most favored regions. Overlapping model showed a correct match between the gB of the two viruses, suggesting a high conserved structure.

### Design and immunoinformatics approach for the vaccine candidate

**Fig. 3:**
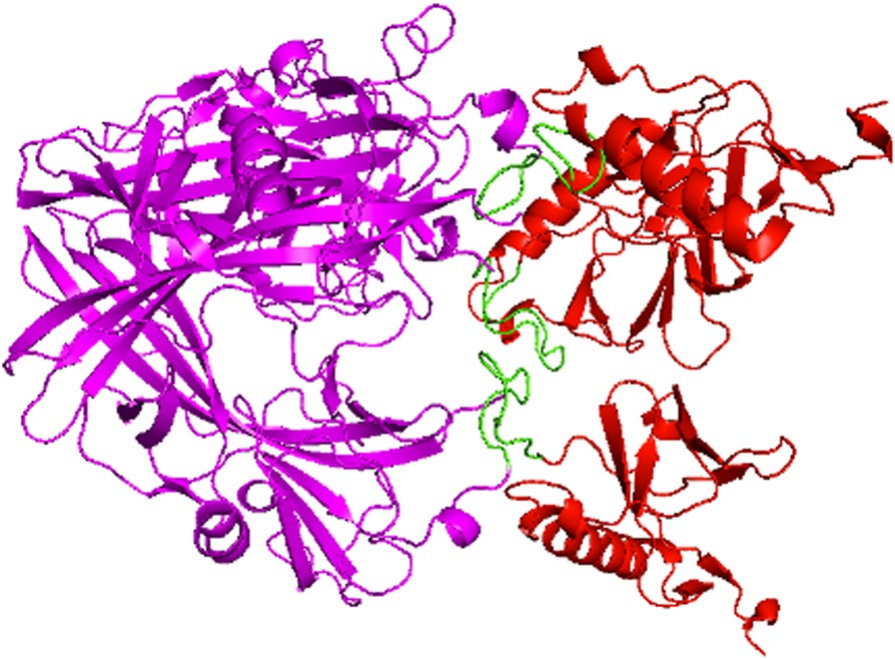
3D Modelling of the vaccine candidate. Magenta—PH-like domain 1 of glycoprotein B of the BoHV-1, Green – Linker GSx8, Red—PH-like domain 2 of glycoprotein B of the BoHV-5

The proposed vaccine candidate is a 355 amino acids protein with a predicted molecular weight of 36.7 kDa. This vaccine candidate contains the PH-like 1 and PH-like 2 domains from BoHV-1 and BoHV-5, respectively, fused through an 8X Gly-Ser linker. Noteworthy, modeling of the tertiary structure of this candidate predicted that the linker allows the correct folding of the domains and their interaction to form a homotrimer with a predicted molecular weight of 110 kDa (Fig. 3).

**Fig. 4:**
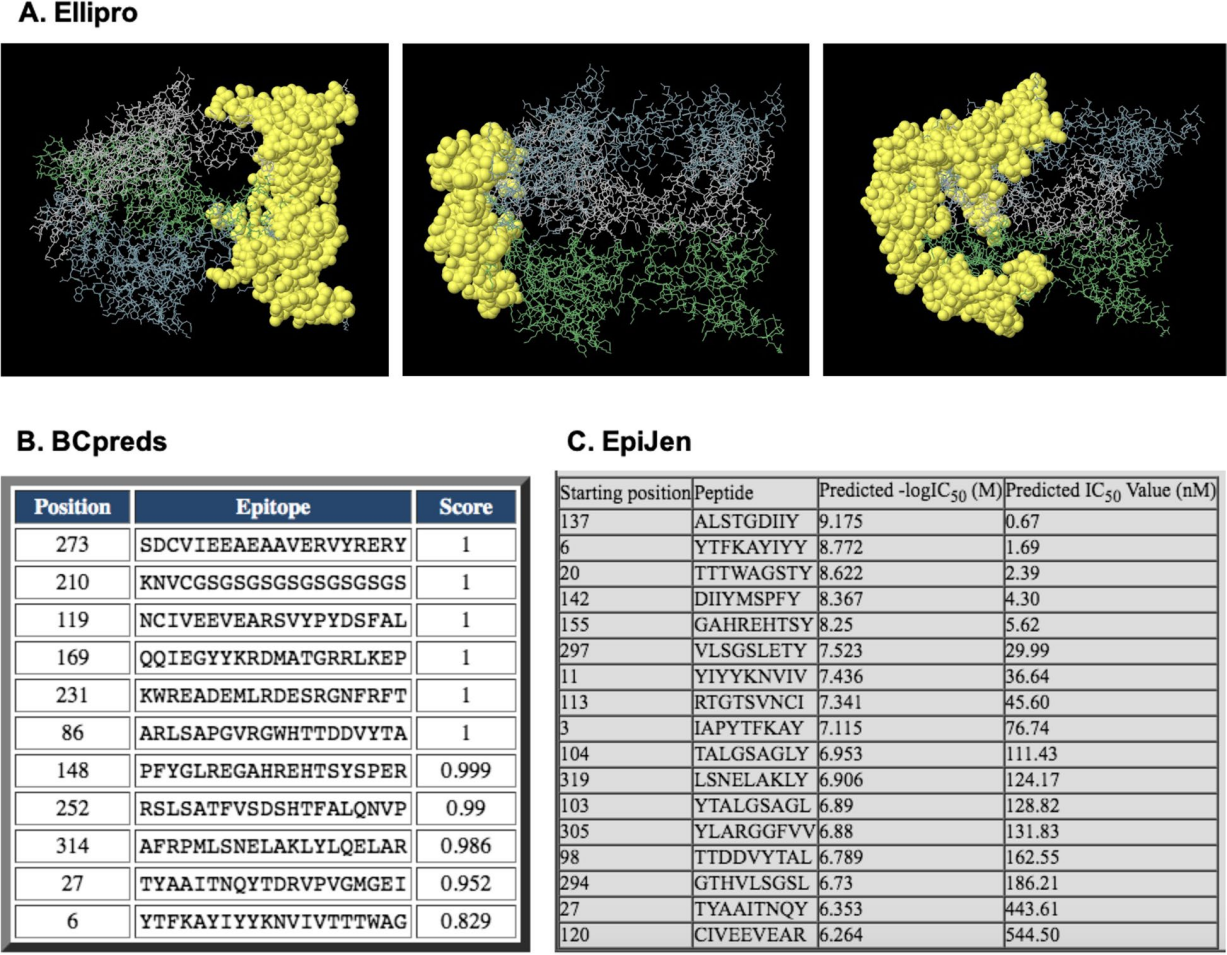
Epitope prediction. **A**. Antibody epitope discontinuous prediction. **B**. Antibody epitope continuous prediction. C. Lymphocyte T epitope prediction

Five discontinuous antibody epitopes of B-lymphocytes with a high-score greater than 0.5 were identified using the Ellipro server. For the case of continuous epitopes of B-lymphocytes, the BCpreds server was used and 11 linear segments were predicted with a high-score ranging from 1–0.8. In addition, using the EpiJen tool we predicted 17 epitopes that can be recognized by TCRs. The resulting epitopes have a considerably important score between 10–6 (Fig. 4).

### Expression, verification and purification of vaccine candidate

**Fig. 5:**
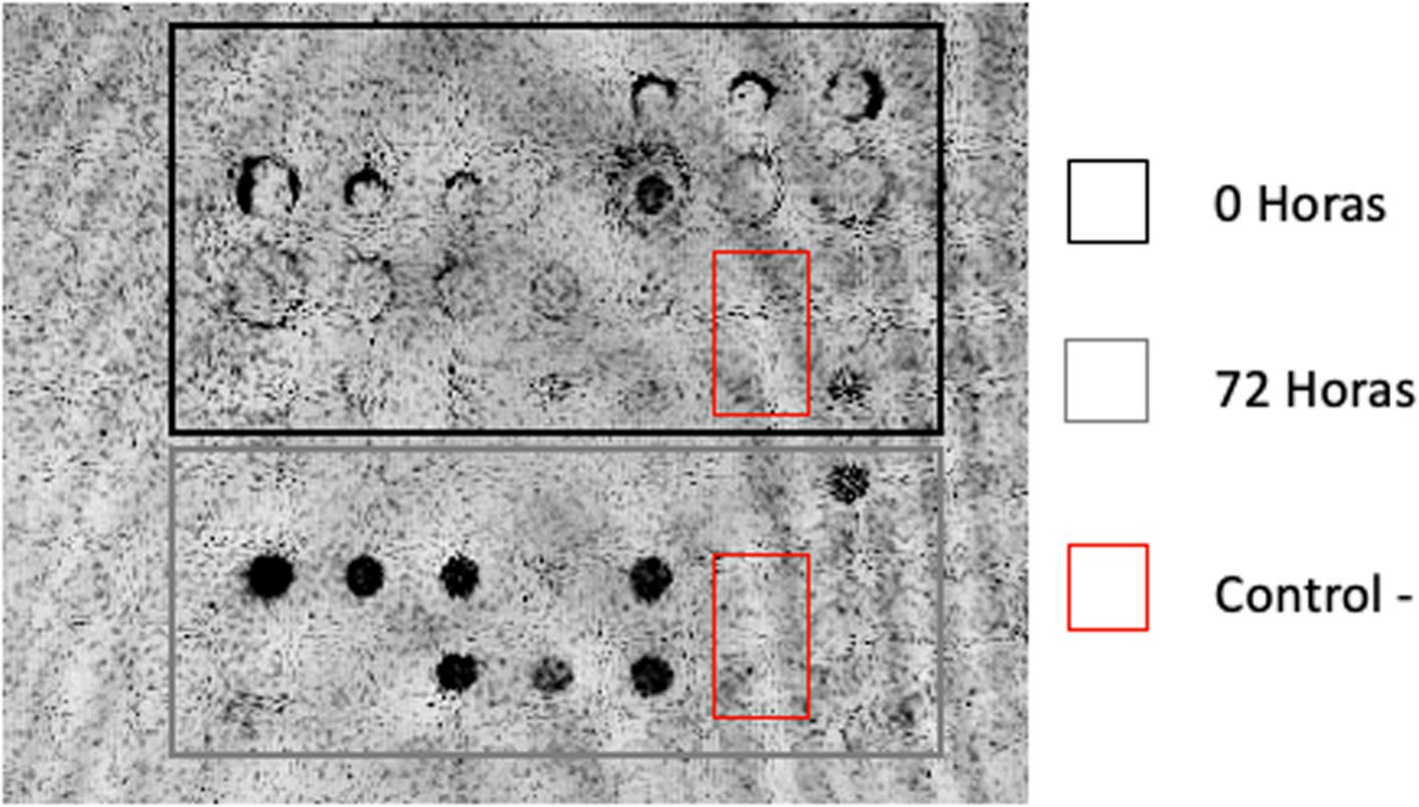
Dot blotting analysis of culture media from transformed *K. phaffii* GS115. Detection of recombinant candidate was performed by using Ingezim IBR 2.0 compact conjugated. Black square indicates 0 h of induction and gray square indicates 72 h of induction. The red square indicates negative clone and the different points are the clones expressing gBDomains. Cropped blot image

Production of recombinant vaccine candidate was performed in *K. phaffii* GS115. Secreted recombinant protein was detected by dot blotting at 72 h post-induction (Fig. 5), which agree with previous reports from the Institute for the Study of Inborn Errors of Metabolism in the production of recombinant proteins in this expression system [23, 24]. As expected, recombinant candidate was not detected with SDS- PAGE in *K. phaffii* GS115 transformed with the empty vector and cultured under the same conditions.

**Fig. 6:**
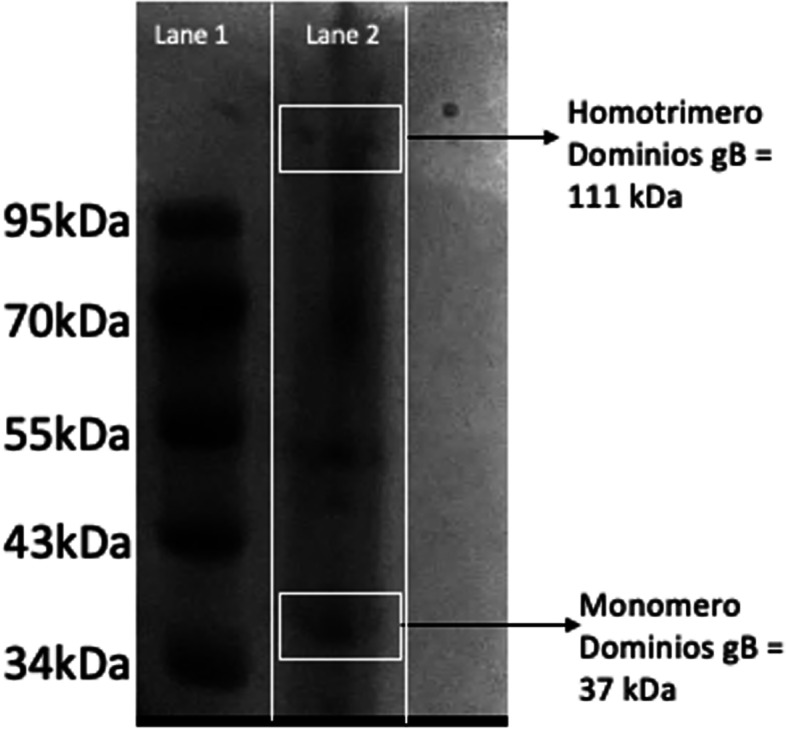
SDS-PAGE of the crude extract from *K. phaffii* GS115 clones. Lane 1: BioPioneer Low Range Pre-Stained Protein Marker, lane 2: gBDomains in reducing and no reducing. Cropped gel image

**Fig. 7:**
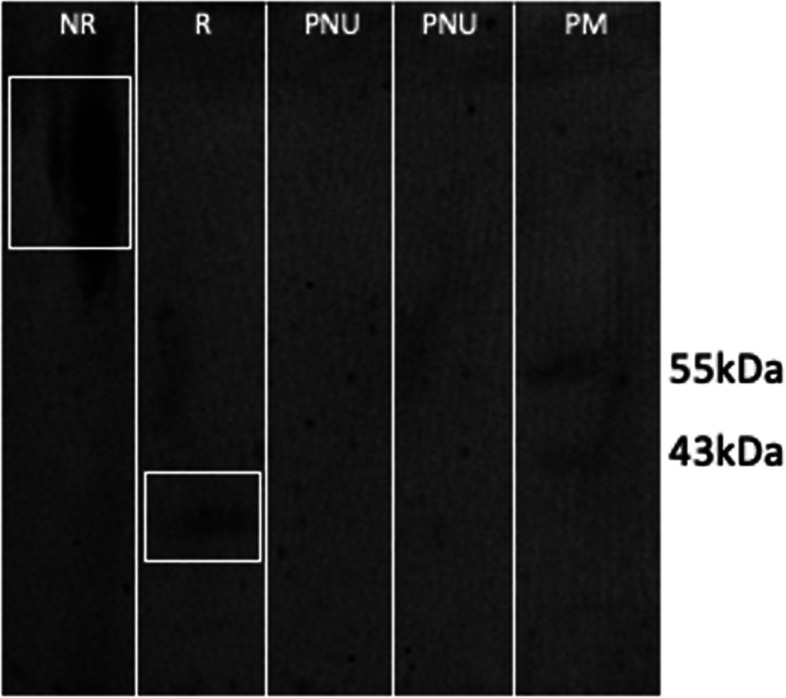
Western blotting analysis of the purification of gBDomains with MAb Anti-6xHis HRP conjugated. Lane 1: Purified recombinant gBDomains in no-reducing condition (NR). Lane 2: Purified recombinant gBDomains in reducing condition (R). Lanes 3–4: unbound protein of purification (PNU). Lane 5. Protein marker (PM). Cropped blot image

The SDS-PAGE of the crude extract shows the presence of two bands (Fig. 6), representing the ∼110 kDa for homotrimer and the ∼37 kDa monomer. Other bands belonging to endogenous proteins of *K. phaffii* were also observed in the SDS-PAGE. Recombinant gB-Domains of purified was detected by using a MAb anti-6xHis HRP conjugated. Bands of ∼110 kDa (no reducing condition) and 37 kDa (reducing condition), were detected through western-blot (Fig. 7). The protein concentration obtained with this process was 873 µg/mL.

## Discussion

In this study, a comparative in silico characterization was carried out between the glycoproteins of BoHV-1 and BoHV-5 using information available in biological databases. The purpose of this study was to identify a glycoprotein that may serve for the design of a bivalent vaccine candidate against BoHV-1 and BoHV-5. With the physicochemical in silico characterization, it was predicted that the different envelope glycoproteins have similar properties among them. Based on the physicochemical characteristics, function, and sequence similarities (Table 1), we selected the gB for further studies [25].

According to the characterization of the primary and secondary structure of gB from the two viral types, the primary structure is highly conserved among these BoHV strains (e-value = 0.0), compared with the other glycoproteins studied. In addition, a high sequence identity was found between these two glycoproteins (91.9%), which suggests a common ancestral strain [23].

The functional annotation of these two glycoproteins predicted the presence of two conserved domains, PH-like 1 and PH-like 2, which may be involved in the binding to the cell receptor (heparan sulfate proteoglycans) during the first steps of the viral cycle [8, 9, 19]. In this sense, directing the humoral immune response through neutralizing antibodies against these domains could prevent virus adhesion and infection [5, 13, 24].

The sequence alignment of BoHV-1/5 B glycoproteins revealed five differences in the PH-like domain 1, while for the PH-like domain 2 eight differences were found. However, the overlapping analysis of the tertiary structures (Fig. 2) of both viruses predicted did not show significant structural differences (RMSD = 0.081) (Fig. 2C). Most of the substitutions observed in these domains were conservative (4 for PH-like domain 1 and 2 for PH-like domain 2) or semi-conservative (3 PH-like domain 2) and only three of them were predicted as severe changes (between Iso-Tre, Glu-Gly and Glu-Ala); in which the residues have different chemical properties.

Based on the results obtained in the bioinformatic study of both gBs, a vaccine candidate based on the PH-Like 1 and Ph-Like 2 domains of these glycoproteins was proposed. First, we performed an in silico immunological analysis of the protein domains, which predicted that these domains could be used in the design of a bivalent vaccine candidate (Fig. 4). The epitopes predicted in regions belonging to both domains of the vaccine candidate suggest a high recognition by the BCRs and TCRs of B and T lymphocytes, respectively [26].

Considering previous studies and the different problems with other prokaryotic organism [20, 27, 28] selected the yeast *K. phaffii* as a host for the recombinant production of the vaccine candidate “gB Domains”. At shaker scale, it was confirmed the expression of the protein of interest (Fig. 5), which was efficiently purified through affinity chromatography (Fig. 7) with final concentration of 873 µg/mL of gBDomains at shaker with 4 cultures of 500 mL. Other studies demonstrated lower purification yields for BoHV glycoproteins using *K. phaffii* expression 190 µg/mL or less [27, 28].

The expression of the vaccine candidate “gB Domains” in *K. phaffii* showed the conservation of the homotrimer in the domains (Fig. 6 and Fig. 7), as it has already happened in previous studies with alphaherpesvirus glycoproteins [27–35]. Noteworthy, the vaccine candidate proposed in this study was recognized by the conjugate antibody from the commercial ELISA kit Ingezim IBR compac 2.0 (Ingenasa). This antibody recognizes glycoprotein B of BoHV-1 (Fig. 5), suggesting that the candidate conserve the conformation and antigenicity of wild-type glycoprotein B from a BoHV viral particle.

## Conclusion

In this paper, we studied the glycoproteins B of BoHV-1 and -5 by using different bioinformatics tools, which allowed to predict that it could be used for the design of a novel potential vaccine candidate. We demonstrated that the combination of the gB domains viruses BoHV1 and 5, within a single protein, could be an immunogenic target for the design of a potential vaccine candidate for IBR. In addition, we produced this vaccine candidate as a recombinant protein in the yeast *K. phaffii*, which facilitate the subsequent scaling up and downstream processes, such as the purifications. Since this novel vaccine candidate was designed as a bivalent vaccine candidate against both types alphaherpesvirus, further studies should evaluate its antigenic capacity. There results support the design of the future in vivo evaluation of the vaccine candidate by using different animal models such as mice, rabbits, and guinea pigs to evaluate the immunogenic potential and the neutralization viral capacity. We consider that in the near future, this vaccine candidate may represent a novel tool against IBR.

## Materials and methods

### Bioinformatic characterization of glycoprotein B

The BoHV-1/5 gB amino acid sequences were retrieved from UniProt (https://www.uniprot.org/). For BoHV-1, Cooper strain (accession numbers P12640) was selected [36] and for BoHV-5 only one reference sequence was available (A0A1Y0B663). ProtParam available in Expasy server [37] was used for prediction of physicochemical properties of the proteins: molecular weight, theoretical isoelectric point (IP), amino acid composition, atomic composition, extinction coefficient, estimated half-life, instability index, aliphatic index, and grand average of hydropathicity (GRAVY).

The conserved domains of the two different gBs were analyzed considering similar sequences based on members of their orthologous family available in several protein databases. CDD-BLAST [38], INTERPROSCAN [39] and Pfam [40] were used for this purpose. PROSITE [41] was used to identify patterns and profiles of the proteins. An individual alignment was carried out between the homologous proteins of BoHV-1 and BoHV-5 using the algorithm CLUSTAL OMEGA available in Mega X [42].

To predict the secondary structure of BoHV1/5 gBs, PSIPRED v3.3 (http://bioinf.cs.ucl.ac.uk/psipred/) was used. The NSP server (https://npsa-prabi.ibcp.fr/cgi-bin/npsa_automat.pl?page=/NPSA/npsa_seccons.html) was used as a secondary test for the prediction of the secondary structure. The tertiary structure of the glycoprotein B of BoHV-1 and BoHV-5 was carried out using a homology-based approach in SWISS PDB Viewer [43] using gB of herpes simplex virus 1 (PDB accession number 5V2S) as a template. The final model was validated by using PROCHECK with Ramachandran plots [44]. Finally, both models were aligned with PyMoL.

### Design and immunoinformatics approach for the vaccine candidate

The PH-Like 1 domain of BoHV-1 gB was fused, via 8X Gly-Ser linker to the PH-Like 2 domain of BoHV-5 gB. The 3D structure of this fused protein was predicted with the same server used. The candidate construct (gBDomains) was evaluated for conformational and linear epitopes for B lymphocytes using Ellipro [45] and BCPreds [46], respectively. Epitopes for T lymphocytes were also predicted using the EpiJen [47] for the candidate construct.

### Expression, verification and purification of vaccine candidate

The recombinant candidate carrying the glycoprotein B domains of BoHV1/5, an 8X Gly-Ser linker, and a C-terminal 6xHis tag was codon optimized for *Komagataella phaffii* GS115 formerly known as *Pichia pastoris* and inserted into pPICZαA expression plasmid (Thermo Fisher Scientific). Recombinant plasmid, pPICZαAgBDomains, was propagated in *E. coli* DH5α, purified with FastGene Plasmid Mini Kit (Genetics), and linearized with *Pme*I (New England Biolabs). Electrocompetent *K. phaffii* GS115 cells were transformed by electroporation with Gene Pulser Xcell Electroporation System (Biorad) (25 μF, 200 Ω, 2 kV) with ∼10 μg of linearized plasmid [48, 49]. Transformed clones were selected in YPD agar (1% yeast extract, 2% peptone, 2% dextrose 2%) supplemented with 1 M sorbitol and 200 μg/mL Zeocin. The plates were incubated at 28 °C for 3 days and sixteen recombinant colonies were selected and replicated in new YPD-200 μg/mL zeocin plates [48, 49].

Screening of the *K. phaffii* clones was done at 10 and 100 mL, as previously reported [23]. Clones were grown in the YPD medium during 48 h at 28 ◦C and 250 rpm. Cells were harvested by centrifugation and incubated in 100 mL of BMGY medium (100 mM potassium phosphate, pH 6.0; 1.34% yeast nitrogen base, 4 × 10^–5^% biotin, 1% glycerol) for 24 h at 28 °C and 250 rpm. Subsequently, the cells were harvested and resuspended in the BMMY medium (100 mM potassium phosphate pH 6.0; 1.34% yeast nitrogen base, 4 × 10^−5^% biotin, 0.5% methanol) and cultured for 72 h at 28 °C and 250 rpm. Methanol was added every 12 h to maintain a final concentration of 0.5% (v/v). Aliquots were taken every 24 h and stored at − 20 ◦C until their use. The clones with the highest protein concentration measured by BCA were selected for further evaluation.

Dot blot analysis was carried out using a nitrocellulose membrane, soaked for 5 min in PBS (pH 7.4) and air-dried. Antigen adsorption was carried out by spotting 50 μL of crude extract of selected *K. phaffii* clones. The membrane was allowed to dry for 1 h and blocked with 5% non-fat powered milk. After washes with wash buffer (IBR Compac 2.0, Ingenasa), the membrane was incubated with an anti-gB conjugated-HRP (IBR Compac 2.0, Ingenasa). Dot blot was visualized by using western Glo Chemiluminescent detection reagents (Thermo Fisher Scientific).

Sodium dodecyl sulfate polyacrylamide gels electrophoresis (SDS–PAGE) was performed as described by Laemmli [50] under reducing conditions. Protein samples (15 μL) were loaded on 12% acrylamide gels and 3% stacking gel in a Mini-PROTEAN electrophoresis system (Bio-Rad). The gel was stained with Coomassie Brilliant Blue R250. For Western blot immunoassay, protein electrophoresis gel was transferred onto a nitrocellulose membrane using Bio-Rad Mini Trans-Blot Cell. The membrane was blocked with 5% non-fat powered milk and antigenic proteins were detected by incubating membrane with MAb Anti-6xHis HRP conjugated (Thermo Fisher Scientific). Membranes were then incubated with anti-mouse immunoglobulins HRP conjugated (1:2000). Specific bands were visualized using enhanced chemiluminescence (SuperSignalTM West Pico Chemiluminescent Substrate, Thermo Fisher Scientific).

The gB domains candidate was obtained from *K. phaffii* culture medium and purified following previously reported methods [48, 49]. Briefly, the crude extract was ultrafiltered through a 10 kDa cutoff membrane (EMD Millipore, Billerica, MA, USA) up to reach a final volume of 50 mL. The retentate was diafiltered against balance buffer (20 mM phosphate buffer, 0.5 mM NaCl, 20 mM imidazol, pH 7,4) and concentrated up to 10 mL by using 10 kDa AmiconR Ultra Centrifugal filter (EMD Millipore Corporation). The gB domains candidate was purified by affinity chromatography using both HisTrap™ HP 1 ml columns pre-packed with pre-charged Ni Sepharose™ and the ÄKTAprime™ Automated Liquid Chromatography System (GE Healthcare). Protein purification was monitored by SDS–PAGE under reducing and not reducing conditions and western blot, as previously described. The protein concentration in the crude extract and purification samples was determined by BCA protein assay (Thermo Fisher Scientific) using bovine serum albumin (BSA) as a standard.

## Supplementary Information

**Additional file 1.**

## Data Availability

The datasets analyzed during the current study are available in Uniprot repository, [BoHV-1, Cooper strain (accession number P12640), BoHV-5 (accession number A0A1Y0B663)]. The datasets analyzed during the current study are available in PDB repository, [glycoprotein B of herpes simplex virus 1 (accession number 5V2S].
