# Supplementary material for: Characterization and expression of domains of Alphaherpesvirus bovine 1/5 envelope glycoproteins B in Komagataella phaffi
Source: BMC Vet Res. 2023 Jan 31;19:28. doi: 10.1186/s12917-023-03590-8 (PMC9887784; doi:10.1186/s12917-023-03590-8)

Figure 5. Dot blotting analysis of culture media from transformed *K. phaffii* GS115. Detection of recombinant candidate was performed by using Ingezim IBR 2.0 compact conjugated. Up points indicate 0h of induction and down points indicates 72h of induction.

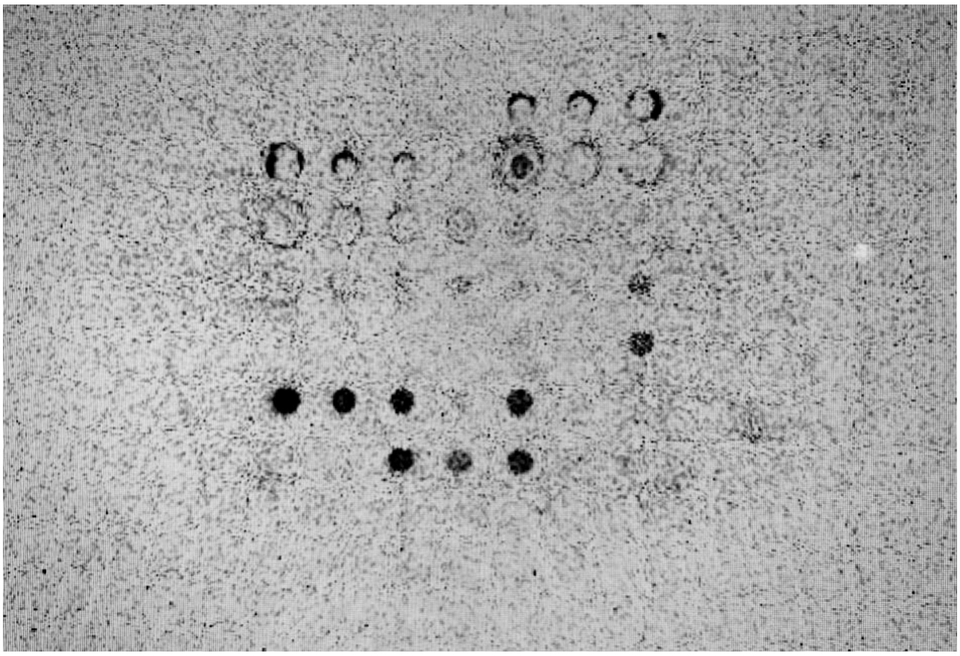

Figure 6. SDS-PAGE of the crude extract from *K. phaffii* GS115 clones. Lane 1: BioPioneer Low Range Pre-Stained Protein Marker, lane 2: gBDomains in reducing and no reducing. Cropped gel image

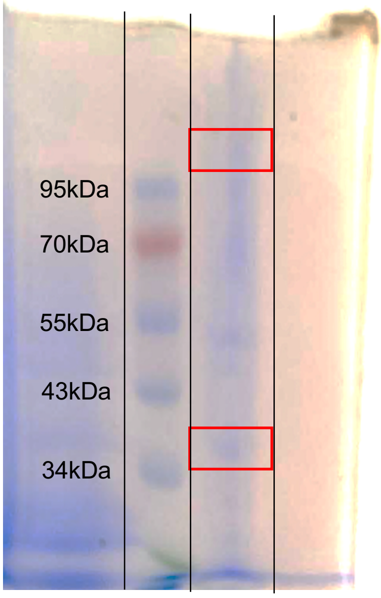

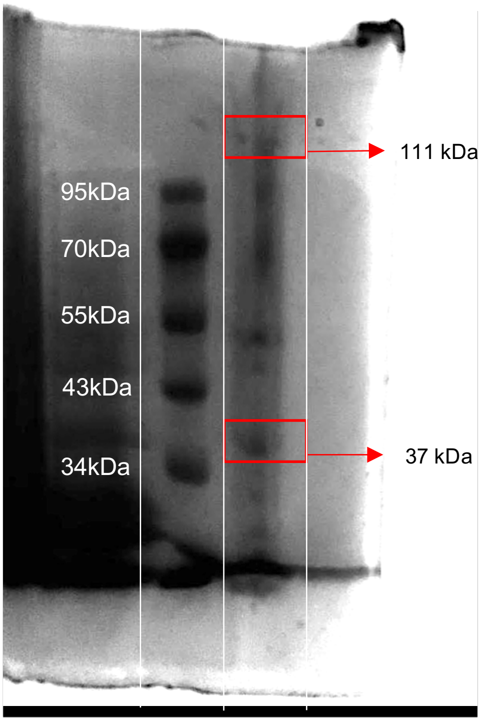

Figure 7. Western blotting analysis of the purification of gBDomains with MAb Anti-6xHis HRP conjugated. Lane 1: Purified recombinant gBDomains in no-reducing condition (NR). Lane 2: Purified recombinant gBDomains in reducing condition (R). Lanes 3-4: unbound protein of purification (PNU). Lane 5. Protein marker (PM). Cropped blot image

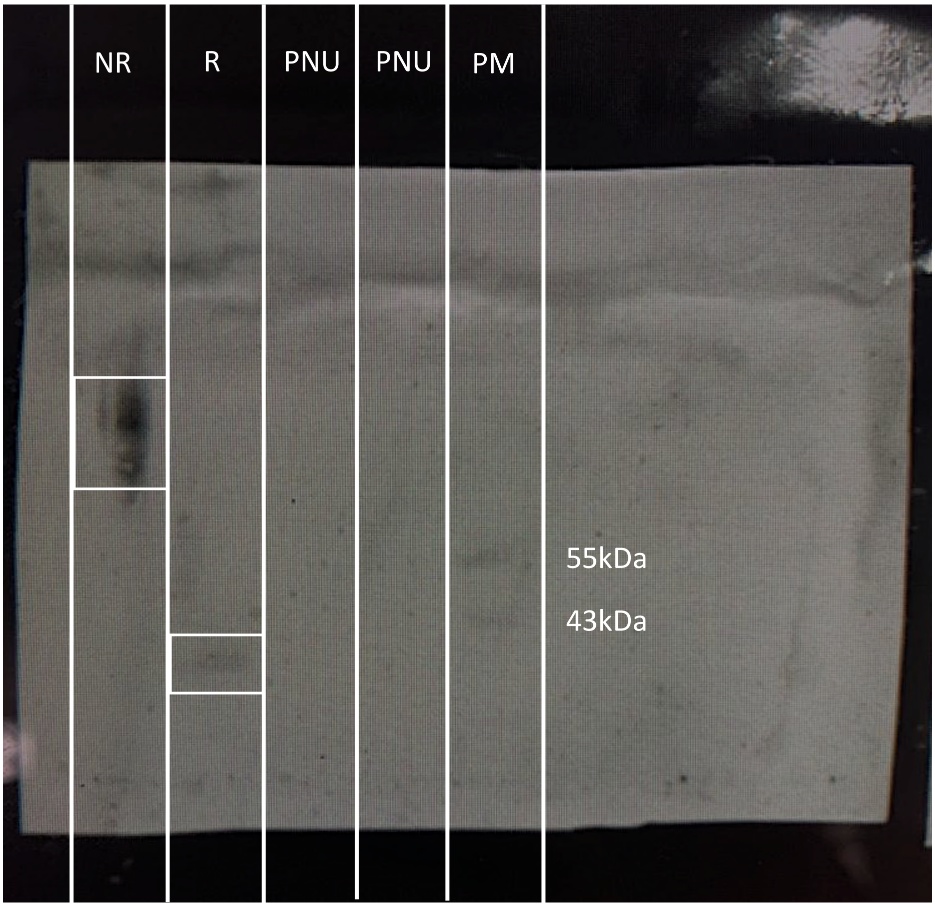

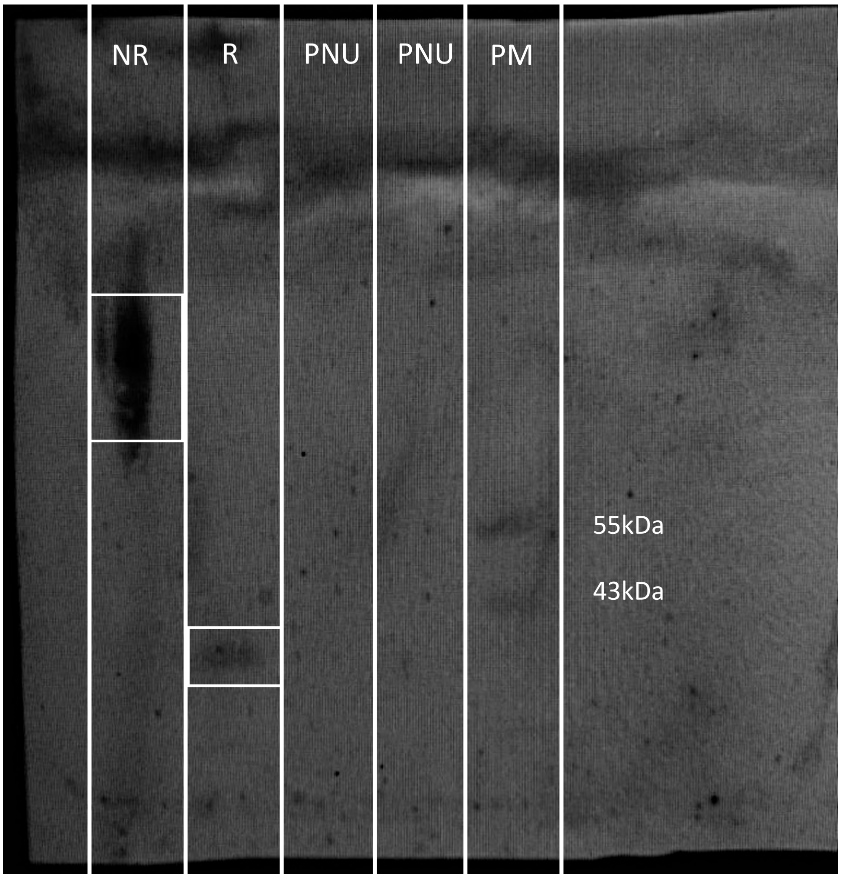

Supplement: Supplementary file 1 — Additional file 1. [file 12917_2023_3590_MOESM1_ESM.pdf]
